# A Global Agenda for Typhoid Control—A Perspective from the Bill & Melinda Gates Foundation

**DOI:** 10.1093/cid/ciy928

**Published:** 2019-02-15

**Authors:** Megan E Carey, Zoey I Diaz, Anita K M Zaidi, A Duncan Steele

**Affiliations:** Enteric and Diarrheal Diseases, Global Health, Bill & Melinda Gates Foundation, Seattle, Washington

## Abstract

Recognizing that enteric fever disproportionately affects the poorest and the most vulnerable communities that have limited access to improved sanitation, safe water sources, and optimal medical care, the Bill & Melinda Gates Foundation has funded efforts to augment global understanding of the disease since the foundation’s inception. At the turn of the century, early efforts focused on characterizing the burden of disease in Asia and evaluating use of the available Vi-polysaccharide vaccines through the Diseases of the Most Impoverished projects at the International Vaccine Institute (IVI). More recent efforts have centered on supporting development of typhoid conjugate vaccines and expanding disease surveillance efforts into Africa, as well as generating a greater understanding of the clinical severity and sequelae of enteric fever in Africa, Asia, and India. The Typhoid Vaccine Accelerator Consortium is playing a critical role in coordinating these and other global efforts for the control of typhoid fever. Here, we outline the scope of support and strategic view of the foundation and describe how, by working through strong partnerships, we can realize a radical reduction of the significance of enteric fever as a global public health problem in the next 10 to 15 years.

After an uncoordinated global approach over the last 2 decades [1], we have seen an unprecedented period of progress in the global public health goal of controlling typhoid fever in the past few years. The acknowledged pathways for long-term control of enteric fever utilizing investments in improved sanitation infrastructure and greater access to safe drinking water, coupled with behavioral changes at the personal and household level, may now be complemented by access to low-cost typhoid conjugate vaccine (TCV). Thus, the appropriate tools are now in hand to affect short- and medium-term control efforts.

Based on the strength of new burden data from Africa and Asia, recent modeled burden estimates indicate that between 11 and 21 million cases of typhoid fever occur annually, with 145,000–161,000 deaths every year [2–4]. These refined estimates, when coupled with promising clinical data of a TCV (Typbar-TCV) developed by Bharat Biotech International Ltd., Hyderabad, India [5], and licensure in India, helped solidify the World Health Organization (WHO) Strategic Advisory Group of Experts’ (SAGE’s) resolve to issue a recommendation for use of new TCVs in children aged >6 months in endemic countries [6]. Shortly thereafter, the WHO prequalified Typbar-TCV, and Gavi, the Vaccine Alliance, approved opening a funding window to support introduction of TCVs in Gavi-eligible countries [7].

Although there is much to celebrate, significant challenges to achieving a true global impact against typhoid remain. Alarming increases in the rates of antimicrobial resistance (AMR), including multidrug-resistant (MDR) strains [8], pose very real threats to typhoid treatment efforts. The threat of preantibiotic era case fatality rates has been heralded by several authors [9]. In the longer term, rapid and ongoing urbanization, global water shortages, and accelerating climate change may limit our ability to control enteric fever through improved water and sanitation interventions and infrastructure development alone.

The global community has a unique opportunity to effectively administer short-term interventions that could support the rapid and sustained reduction of typhoid fever as a public health problem in some of the most vulnerable areas of the world. It is critical that new epidemiology and burden data and evidence of the increasing threat of antimicrobial resistance be communicated to policy-makers in-country in order to facilitate informed decision-making regarding TCV introduction. Equally important will be monitoring and evaluation activities geared toward the generation of consolidated evidence from the first countries that use TCVs at scale. Lessons learned from such efforts will ensure that country introduction strategies are designed and implemented appropriately.

## ENHANCED UNDERSTANDING OF THE BURDEN OF DISEASE

Several multicenter hospital-based surveillance studies have enhanced our understanding of the global burden of typhoid fever in recent years [10–12]. Additional data are forthcoming that will further refine our understanding of age and geographic distribution of the disease, as well as patterns of antimicrobial resistance.

In recognition of the paucity of *Salmonella* burden data from Africa, a passive surveillance system for detection of bloodstream infections among febrile patients at sentinel sites in 10 African countries (Burkina Faso, Ethiopia, Ghana, Guinea-Bissau, Kenya, Madagascar, Senegal, South Africa, Sudan, and Tanzania) was established by the IVI in 2009 [10, 13]. This demonstrated that typhoid fever incidence varied widely across the continent, although the observed overall incidence of typhoid fever in Africa was 2–3 times higher than previously thought [10, 13, 14], with the highest burden occurring in children aged 2–5 years (adjusted incidence 191.8 per 100,000 person-years; 95% confidence interval, 51–721.2) [10]. In addition, high incidence of typhoid fever was observed both in densely populated urban areas as well as less densely populated rural areas. Furthermore, almost half (47%) of *Salmonella* Typhi isolates cultured in this study exhibited multidrug resistance. A follow-on study was initiated in 2016 to document the burden of severe typhoid and associated sequelae as well as to generate the updated cost of illness data and further elucidate AMR profiles; the study is currently enrolling in 6 countries (Burkina Faso, Ghana, Madagascar, Ethiopia, Democratic Republic of Congo, and Nigeria).

While the magnitude of the burden of enteric fever in South Asia has been described in studies conducted 2 decades ago [15], questions remain about the age-specific incidence, mortality, clinical complications, hospitalization rates, AMR prevalence, and economic impact of disease. The Sabin Institute established a multicountry, multisite surveillance study aimed at answering these questions at several South Asian sites. Initially, this consisted of a 2-year retrospective review of existing data, which showed consistently high rates of blood cultures that were confirmed as *S* Typhi or Paratyphi in Bangladesh, India, Nepal, and Pakistan, as described in this issue of *Clinical Infectious Diseases* [16]. Currently, a prospective hospital-based surveillance study is generating new incidence rates and is looking at clinical features of severe disease, long-term sequelae, antimicrobial resistance patterns, and cost of illness in Bangladesh, Nepal, and Pakistan. The study uses a hybrid approach to passive surveillance, pairing facility-based surveillance with population-based healthcare utilization surveys to estimate population denominators [17].

There has been little recent nationally representative typhoid incidence data published from India, as highlighted in a recent systematic review of typhoid burden studies [18]. Recognition of this data gap led to establishment of the National Surveillance System for Enteric Fever in India (NSSEFI), which was developed in concert with the Indian Council of Medical Research, the Translational Health Sciences and Technology Institute, Indian Academy of Pediatrics, and other stakeholders. NSSEFI, which was started in late 2017, has established 3 tiers of typhoid surveillance in India [19], including active, community-based surveillance in 4 geographically representative sites; passive, hospital-based surveillance in 6 hospitals that use a hybrid approach similar to that described above; and laboratory-based surveillance, which is being conducted at another 8 sites to generate country-wide data critical to a fuller understanding of the true burden of typhoid fever in India. It is expected that these data will ultimately be useful to the National Technical Advisory Group on Immunization in deciding if, and where, TCV introduction should be prioritized in India.

*Salmonella* Typhi strains that bear AMR phenotypes have been recorded over the past 4 decades and have systematically led to the switch of antibiotics used in various regions. However, the global spread of MDR strains has accelerated and is now commonly identified in Asia and Africa where disease burden is highest and resources are limited. MDR *S*. Typhi has been reported in Asia [11, 20], the Middle East [21], and sub-Saharan Africa [22, 23]. *Salmonella* Typhi haplotype H58 has become the dominant strain in many regions and is highly associated with increased multidrug resistance and rapid spread of these strains [24, 25]. These MDR H58 haplotypes are increasingly resistant to fluoroquinolones [25] and are aggressively displacing other bacterial lineages. Alarmingly, H58 strains with simple *gyrA* mutations are thought to have a biological competitive advantage, even in the absence of antimicrobial therapy [26]. A recent outbreak of *S.* Typhi strains resistant to all first-line drugs (ie, chloramphenicol, ampicillin, and trimethoprim-sulfamethoxazole) as well as fluoroquinolones and third-generation cephalosporins in Pakistan [8] has caused considerable concern as the harbinger of what is to come.

Despite the new epidemiological data and sobering views on worsening rates of multidrug resistance, there is still a paucity of blood culture surveillance data available in many countries, given the time, cost, and technical skill required to perform blood cultures [27]. This raises questions for decision-makers about what types of data are sufficient to support a decision to introduce the vaccine. Even where blood culture is established, the above-mentioned limitations coupled with low sensitivity (50%–60%) [27, 28] mean that many febrile patients are treated empirically, leading to early, widespread, and often unwarranted antibiotic usage, which has implications for the continued spread of resistance. Considering these difficulties, there continues to be the need for a simple, highly sensitive, nonresource-intensive, rapid diagnostic for clinical use. Furthermore, recognizing the limitations of current national clinical data on typhoid disease, we are exploring the potential utility of environmental surveillance to explore subregional or district-level typhoid circulation.

## TYPHOID CONJUGATE VACCINE PERFORMANCE

We have seen rapid, successful progression of a TCV (Typbar-TCV) as a potential global public health tool, since these authors drafted a “call to action” in 2016 [1]. Typbar-TCV was licensed in India based on robust immunogenicity and GMT data [5], which enabled comparison of immunogenicity to that observed in field studies with the earlier US National Institutes of Health Vi-rEPA TCV tested in young children in Vietnam [29]. To strengthen the clinical data for Typbar-TCV, the vaccine was evaluated in the Oxford Vaccine Group’s controlled human infection model for typhoid fever. The study showed 87.1% efficacy against typhoid fever in immunologically naive patients using a clinical definition of disease (fever ≥38.0°C followed by positive *S.* Typhi blood culture) [30]. This level of protection is similar to that observed in the Vietnamese field trials in children aged 2–5 years [29]. The WHO SAGE recommendation and WHO prequalification were both supported by the strength of this additional data.

Subsequent reanalysis of long-term immunogenicity data from the Typbar-TCV phase 3 randomized, controlled trial further strengthens the case for the significant potential impact of TCVs [31]. By modeling anti-Vi immunoglobulin G antibody decay that would be expected at 42, 540, and 720 days post-vaccine and comparing those curves to immunogenicity curves from patients in the Oxford study, one could assume that significant deviation from expected antibody kinetics represented natural typhoid infection; these rates of infection were compared in the Vi-TT and Vi-PS arms, assuming 59% vaccine efficacy of Vi-PS. This yielded a 2-year “sero-efficacy” estimate of 85%, which is also comparable to the field efficacy from the early Vi-rEPA field studies as well as the efficacy against clinical disease observed in the CHIM study referenced above.

Finally, the Typhoid Vaccine Acceleration Consortium (TyVAC), a consortium between the University of Maryland, Oxford University, and PATH, is assessing the impact of this vaccine when deployed at scale in endemic countries. TyVAC is currently conducting 3 large field efficacy studies using Typbar-TCV in Bangladesh, Nepal, and Malawi [32]. The studies will generate important data on the evidence of TCV impact in endemic settings to further advance our understanding of its potential role. TyVAC is also engaged in broader global and country awareness and policy and has a broad mandate for coordination and advocacy across the field.

## TYPHOID CONJUGATE VACCINE DEVELOPMENT PIPELINE

To promote supply security and price competition, it is important to have more than 1 supplier for public markets. While there is currently only 1 TCV prequalified and available to Gavi countries, several other TCV candidates are in clinical development. Some of these vaccine candidates have different conjugation technologies and carrier proteins but should confer levels of protection similar to those of Typbar TCV and will provide additional global supply of low-cost vaccines for public markets.

IVI has developed a Vi-polysaccharide conjugated to diphtheria toxoid (Vi-DT) vaccine construct [33] that has been tech-transferred to SK Chemicals (now SK Bioscience) in South Korea, PT BioFarma in Indonesia, and Incepta Vaccine Ltd. in Bangladesh. A randomized, observer-blind, age-descending phase 1 study to assess the safety and immunogenicity of the Korean Vi-DT vaccine was completed in Manila recently. The study showed that the product was safe and immunogenic [34]. A randomized, observer-blinded phase 2 safety, reactogenicity, and immunogenicity study in healthy infants and toddlers (aged 6–23 months) is ongoing in the Philippines. Similarly, the PT BioFarma has completed a randomized, observer-blinded phase I safety and immunogenicity study of Vi-DT in Indonesian adults and children in February 2018, and results are being prepared for publication.

An alternative typhoid Vi-polysaccharide conjugated to CRM_197_ construct has been developed by Global Vaccines for Global Health, Sienna (originally Novartis Vaccine Institute for Global Health) [35, 36]. Biological-E, Hyderabad, India, is continuing the development of this candidate and completed a phase 1 study in adults in India (CTRI/2018/03/012558). Results are not yet published, and plans for subsequent clinical development are still under discussion.

These candidates are likely to progress to WHO prequalification and, with Gavi funding, provide an expanded opportunity for countries to introduce life-saving, cost-effective vaccines. Decisive action from decision-makers in-country to introduce and scale up TCVs will not only prevent infections and save lives in those settings but will also incentivize additional manufacturers to enter the market and operate at higher production capacities, which should lead to greater supply security and generate price competition.

## REMAINING CHALLENGES FOR TYPHOID CONTROL

This is an important time for aggressive pursuit of typhoid control, given the unprecedented global momentum. However, there continues to be a need to better understand typhoid transmission dynamics and how these vary both temporally and geographically in order to more accurately guide intervention strategies. In areas where laboratory-confirmed burden data are limited, there is a need to establish feasible ways to utilize existing surveillance data and/or proxy measures of burden (eg, modeled data) in order to inform country decisions on optimal TCV delivery strategies. Additionally, we need a better understanding of the outbreak potential of typhoid and optimal vaccine response strategies to effectively intervene. Finally, and ominously, the increasing evidence and spread of MDR strains threatens to significantly increase typhoid case fatality rates, potentially to preantibiotic levels. Thus, it is imperative that we understand what barriers exist for country-level TCV introduction and ensure broad awareness of the burden of typhoid and the rationale for intervention in endemic settings.

We are encouraged not only by recent data and policy recommendations but also by the renewed energy and collaborative spirit that have taken hold in the global typhoid community. If the strong partner ecosystem that has been developed maintains this level of momentum, we should be well positioned to tackle these challenges in the years ahead.

## Notes

***Potential conflicts of interest.*** All authors: no reported conflicts. All authors have submitted the ICMJE Form for Disclosure of Potential Conflicts of Interest. Conflicts that the editors consider relevant to the content of the manuscript have been disclosed.
